# Lenvatinib in patients with unresectable hepatocellular carcinoma who progressed to Child-Pugh B liver function

**DOI:** 10.1177/17588359221116608

**Published:** 2022-08-24

**Authors:** Jasmine Huynh, May Thet Cho, Edward Jae-Hoon Kim, Min Ren, Zahra Ramji, Arndt Vogel

**Affiliations:** University of California Davis Comprehensive Center, Sacramento, CA, USA; University of California Irvine Health, Orange, CA, USA; University of California Davis Comprehensive Center, Sacramento, CA, USA; Eisai Inc., Nutley, NJ, USA; Eisai Inc., Nutley, NJ, USA; Hannover Medical School, Carl-Neuberg- Straße 1, Hannover 30625, Germany

**Keywords:** Child-Pugh B, hepatocellular carcinoma, lenvatinib, liver impairment, systemic treatment

## Abstract

**Background:**

Lenvatinib is an approved first-line treatment for unresectable hepatocellular carcinoma (uHCC). We evaluated the safety and efficacy of lenvatinib *versus* sorafenib in patients with uHCC who deteriorated to Child-Pugh class B (CP-B) on treatment.

**Methods:**

We retrospectively evaluated patients from REFLECT who deteriorated to CP-B *versus* those who remained Child-Pugh class A (CP-A) within 8 weeks after randomization. Best overall response and objective response rate (ORR) per modified Response Evaluation Criteria In Solid Tumors (mRECIST) were assessed from baseline. Progression-free survival (PFS) per mRECIST and overall survival (OS) were assessed beginning at week 8.

**Results:**

Patients with CP-B *versus* CP-A classification receiving lenvatinib had ORRs of 28.3 and 42.9%, respectively; patients with CP-B *versus* CP-A classification receiving sorafenib had ORRs of 8.5 and 12.9%, respectively. Median PFS and OS (landmark analyses beginning at week 8) in patients receiving lenvatinib were 3.7 months [95% confidence interval (CI): 1.8–7.4] and 6.8 months (95% CI: 2.6–10.3) in the CP-B subgroup *versus* 6.5 months (95% CI: 5.6–7.4) and 13.3 months (95% CI: 11.6–16.1) in the CP-A subgroup, respectively. Median PFS and OS in patients receiving sorafenib were 0.5 months (95% CI: 0.1–3.6) and 4.5 months (95% CI: 2.9–6.1) in the CP-B subgroup *versus* 3.6 months (95% CI: 2.7–3.7) and 12.0 months (95% CI: 10.2–14.0) in the CP-A subgroup, respectively. The most common treatment-emergent adverse events in the lenvatinib cohort were hypertension (both subgroups) and decreased appetite (CP-B subgroup).

**Conclusion:**

Results suggest that patients with uHCC whose liver function deteriorates to CP-B after initiation of therapy may continue to receive lenvatinib.

**Trial registration:**

ClinicalTrials.gov, NCT01761266, https://clinicaltrials.gov/ct2/show/NCT01761266.

Hepatocellular carcinoma (HCC) is the most common form of liver cancer, accounting for about 90% of cases,^1^ with a dismal 5-year survival rate of 18%.^2^ The main risk factor for HCC is liver cirrhosis mainly caused by nonalcoholic fatty liver disease, alcohol exposure, or infection by hepatitis B or C viruses.^1^ Patients with Child-Pugh class B (CP-B) liver function are frequently excluded from clinical trials due to the severity of their underlying liver disease,^3,4^ and the National Comprehensive Cancer Network guidelines recommend most systemic therapy options for CP class A (CP-A) patients only.^5^ Thus, there is a lack of information about the safety and efficacy of systemic treatment for these patients.^6^ Lenvatinib, a multikinase inhibitor of vascular endothelial growth factor receptors 1–3, fibroblast growth factor receptors 1–4, platelet-derived growth factor receptor α, RET, and KIT,^7–11^ is approved as a first-line treatment for patients with unresectable HCC (uHCC) based on the phase III, randomized, multicenter, REFLECT study, which evaluated lenvatinib *versus* sorafenib.^12^ In REFLECT, the survival time for patients with uHCC treated with lenvatinib (median 13.6 months, 95% confidence interval [CI]: 12.1–14.9) was noninferior to sorafenib (median 12.3 months, 95% CI: 10.4–13.9). Per the inclusion criteria, patients should have had CP-A liver function at baseline.

In a prior analysis of REFLECT, patients treated with lenvatinib experienced a benefit, irrespective of baseline liver function [albumin–bilirubin (ALBI) grade 1 or 2; Child-Pugh (CP) score 5 or 6].^13^ The objective response rate (ORR) was higher with lenvatinib *versus* sorafenib in patients with either a CP score of 5 (odds ratio: 4.88; 95% CI: 3.37–7.08) or a CP score of 6 (odds ratio: 5.25; 95% CI: 2.32–11.85). ORR was also higher with lenvatinib *versus* sorafenib in patients classified as either ALBI grade 1 (odds ratio: 5.48; 95% CI: 3.70–8.10) or ALBI grade 2 (odds ratio: 5.37; 95% CI: 2.61–11.06). In addition, median overall survival (OS) was longer with lenvatinib *versus* sorafenib in patients classified as either ALBI grade 1 (17.4 months *versus* 14.6 months; hazard ratio [HR], 0.85 [95% CI: 0.70–1.02]) or ALBI grade 2 (8.6 months *versus* 7.7 months; HR, 0.95 [95% CI: 0.73–1.25]).

Observational transarterial chemoembolization (TACE) studies report liver function deterioration after treatment of patients with HCC.^14–16^ Additional rounds of TACE are contraindicated for individuals whose liver function deteriorates as a result of treatment.^17^ Similarly, post hoc analyses of clinical trials with systemic therapies in patients with advanced HCC have shown that liver function may deteriorate during treatment.^4,18^ However, a retrospective analysis of the randomized phase III CELESTIAL trial of patients with advanced HCC who received cabozantinib showed that those who deteriorated from CP-A to CP-B liver function by week 8 could still experience efficacy with continued treatment.^4^ Dose reduction and discontinuation rates in the CP-B subgroup were comparable to the overall population. Taken together, these results suggest that despite frequent exclusion of patients with CP-B liver function classification from studies of systemic therapies,^3^ these treatments may be effective and tolerable in patients whose liver function deteriorates during treatment. However, stronger evidence for systemic therapies in this patient population is lacking as few studies have examined the relationship between efficacy and changes in liver function. In general, there are multiple possible reasons for deterioration of liver function, including tumor progression, underlying liver disease, or treatment-related toxicity. Studies that can help clinicians make decisions on how and whether to continue treating patients with uHCC and liver function deterioration during treatment are limited. As such, we conducted a post hoc exploratory analysis of key efficacy and safety outcomes in patients from REFLECT whose liver function deteriorated to CP-B (CP-B subgroup) *versus* those whose liver function remained CP-A (CP-A subgroup) within 8 weeks after randomization to gain insight into the safety and efficacy outcomes of patients who continued treatment after deterioration of liver function to CP-B.

## Methods

### REFLECT trial details: patients, study design, and treatments

REFLECT was an open label, phase III, noninferiority trial that compared lenvatinib *versus* sorafenib as a first-line treatment for patients with uHCC. Full details of the study have been published.^12^ Briefly, eligible patients had uHCC with ⩾1 measurable target lesion per modified Response Evaluation Criteria In Solid Tumors (mRECIST), a Barcelona Clinic Liver Cancer (BCLC) stage B or C, an Eastern Cooperative Oncology Group performance status (ECOG PS) score ⩽1, and CP-A liver function classification. Patients were excluded if they had previous systemic therapy for HCC, HCC with ⩾50% liver occupation, clear invasion into the bile duct, or main portal branch invasion (Vp4).

Patients were randomly assigned 1:1 to receive lenvatinib (*n* = 478) or sorafenib (*n* = 476). Stratification was based on region (Asia-Pacific or Western), macroscopic portal vein invasion, extrahepatic spread, or both (yes or no), ECOG PS (0 or 1), and bodyweight (<60 or ⩾60 kg). Study drugs were administered in 28-day cycles. Lenvatinib starting dose was based on patients’ bodyweight – lenvatinib 12 mg/day for patients who weighed ⩾60 kg and lenvatinib 8 mg/day for those who weighed <60 kg. Sorafenib was administered at 400 mg twice daily.

### Post hoc subgroup analyses

This post hoc exploratory subgroup analysis included patients from REFLECT. Patients were divided into subgroups based on liver function. The CP-B group included patients whose liver function had deteriorated to CP-B within 8 weeks post-randomization, and the CP-A subgroup included patients from REFLECT whose liver function remained CP-A within 8 weeks post-randomization (the 8-week timepoint was chosen to align with the CELESTIAL trial post-hoc analysis of patients with advanced HCC who progressed to CP-B during treatment with cabozantinib).^4^ Best overall response and ORR were assessed from baseline; progression-free survival (PFS), and OS were assessed using landmark analyses (that began at week 8) for lenvatinib-treated and sorafenib-treated patients in the CP-B and CP-A subgroups. The ORR analysis included all patients in the CP-B and CP-A subgroups; due to the landmark cutoff, PFS and OS analyses included fewer patients, as patients who did not reach the landmark were not included. Tumors were assessed by independent imaging review (IIR) per mRECIST.

### Statistical methods

Landmark analyses (that began at week 8) of PFS and OS in the CP-B and CP-A subgroups were performed using the Kaplan–Meier method, and 95% CIs of median PFS and OS were calculated using a generalized Brookmeyer and Crowley method. Safety was evaluated from baseline and adjusted by treatment duration. Adverse events (AEs) were graded using Common Terminology Criteria for Adverse Events version 4.0.

All authors had access to the study data and had reviewed and approved the final manuscript.

## Results

### Baseline characteristics of patients

The data cutoff for analyses reported herein is 13 November 2016 (the cutoff used in the main REFLECT study).^12^ Baseline demographic and disease characteristics for patients in the CP-B and CP-A subgroups within the lenvatinib and sorafenib treatment arms are shown in Table 1. Among the 60 patients in the lenvatinib arm whose liver function deteriorated to CP-B within 8 weeks, 44 (73.3%) had a baseline ALBI score of 2 and 39 (65.0%) had a baseline CP score ⩾6. In the sorafenib arm, 29 (61.7%) and 26 (55.3%) of the 47 patients in the CP-B subgroup had a baseline ALBI score of 2 and a baseline CP score of ⩾6, respectively. In the subgroup of patients whose liver function remained CP-A within 8 weeks, 302 (73.1%) and 345 (83.5%) patients in the lenvatinib arm (*n* = 413); and 322 (75.4%) and 335 (78.5%) patients in the sorafenib arm (*n* = 427), had a baseline ALBI score of 1 and a baseline CP score of 5, respectively. In the lenvatinib arm, the median percent changes in the sum of tumor diameters from baseline to week 8 (before the initiation of landmark analyses for PFS and OS) were –16.2% (range –100 to 25) in the CP-B subgroup and –15.9% (range –100 to 187) in the CP-A subgroup, respectively, whereas in the sorafenib arm, these numbers were 1.7% (range –36 to 64) and 0% (range –100 to 106) in the CP-B and CP-A subgroups, respectively.

**Table 1.** Baseline and disease characteristics.

Category	Lenvatinib		Sorafenib	
	CP-B subgroup^a^, *n* = 60	CP-A subgroup^b^, *n* = 413	CP-B subgroup^a^, *n* = 47	CP-A subgroup^b^, *n* = 427
Median age, years (range)	64.0 (34–86)	63.0 (20–88)	64.0 (26–79)	62.0 (22–88)
Sex, *n* (%)				
Male	45 (75.0)	355 (86.0)	38 (80.9)	362 (84.8)
Female	15 (25.0)	58 (14.0)	9 (19.1)	65 (15.2)
Body weight, *n* (%)				
<60 kg	21 (35.0)	132 (32.0)	16 (34.0)	128 (30.0)
⩾60 kg	39 (65.0)	281 (68.0)	31 (66.0)	299 (70.0)
ECOG performance status, *n* (%)				
0	40 (66.7)	264 (63.9)	18 (38.3)	283 (66.3)
1	20 (33.3)	149 (36.1)	29 (61.7)	144 (33.7)
Factor of carcinogenesis, *n* (%)				
Hepatitis B	29 (48.3)	219 (53.0)	20 (42.6)	206 (48.2)
Hepatitis C	15 (25.0)	75 (18.2)	12 (25.5)	114 (26.7)
Alcohol	7 (11.7)	28 (6.8)	3 (6.4)	18 (4.2)
Other	6 (10.0)	32 (7.7)	3 (6.4)	29 (6.8)
Unknown	3 (5.0)	59 (14.3)	9 (19.1)	60 (14.1)
Median sum of tumor diameters, mm (range)	69.5 (11–225)	59.6 (10–284)	77.2 (13–273)	60.5 (10–283)
ALBI score, *n* (%)				
1	16 (26.7)	302 (73.1)	18 (38.3)	322 (75.4)
2	44 (73.3)	111 (26.9)	29 (61.7)	104 (24.4)
3	0	0	0	1 (0.2)
BCLC stage, *n* (%)				
B	14 (23.3)	89 (21.5)	2 (4.3)	90 (21.1)
C	46 (76.7)	324 (78.5)	45 (95.7)	337 (78.9)
CP score, *n* (%)				
5	21 (35.0)	345 (83.5)	21 (44.7)	335 (78.5)
6	36 (60.0)	68 (16.5)	21 (44.7)	92 (21.5)
7	3 (5.0)	0	4 (8.5)	0
8	0	0	1 (2.1)	0
AFP level ⩾200 ng/mL, *n* (%)	33 (55.0)	186 (45.0)	25 (53.2)	161 (37.7)
Macroscopic portal vein invasion, extrahepatic spread, or both, *n* (%)				
Yes	45 (75.0)	280 (67.8)	38 (80.9)	297 (69.6)
No	15 (25.0)	133 (32.2)	9 (19.1)	130 (30.4)
Underlying cirrhosis^c^, *n* (%)				
Yes	54 (90.0)	298 (72.2)	39 (83.0)	324 (75.9)
No	6 (10.0)	115 (27.8)	8 (17.0)	103 (24.1)

a Includes patients who deteriorated to CP-B liver function within 8 weeks post-randomization.

b Includes patients with CP-A liver function within 8 weeks post-randomization.

c By IIR.

AFP, alpha-fetoprotein; ALBI, albumin–bilirubin; BCLC, Barcelona Clinic Liver Cancer; CP, Child-Pugh; ECOG, Eastern Cooperative Oncology Group; IIR, independent imaging review.

### Efficacy

In the lenvatinib arm, the ORRs by IIR per mRECIST were 28.3% (95% CI: 16.9–39.7) and 42.9% (95% CI: 38.1–47.6) in the CP-B and CP-A subgroups, respectively (Table 2). In the CP-B subgroup, no patients achieved a complete response (CR) and 17 (28.3%) patients had a partial response (PR). In the CP-A subgroup, 10 (2.4%) and 167 (40.4%) patients had a CR and PR, respectively. The median time to first objective response was 1.9 months in both the CP-B subgroup (range 2–13) and the CP-A subgroup (range 1–15). In the sorafenib arm, the ORR was 8.5% (95% CI: 0.5–16.5) and 12.9% (95% CI: 9.7–16.1) in the CP-B and CP-A subgroups, respectively (Table 2). In the CP-B subgroup, no patients had a CR and 4 (8.5%) patients had a PR. In the CP-A subgroup, 4 (0.9%) patients had a CR and 51 (11.9%) patients had a PR. The median time to first objective response was 2.7 months (range 2–9) in the CP-B subgroup and 2.1 months (range 2–15) in the CP-A subgroup.

**Table 2.** Efficacy outcomes per IIR by mRECIST.

Parameter	Lenvatinib		Sorafenib	
	CP-B subgroup, *n* = 60	CP-A subgroup, *n* = 413	CP-B subgroup, *n* = 47	CP-A subgroup, *n* = 427
Best overall response, *n* (%)				
Complete response	0	10 (2.4)	0	4 (0.9)
Partial response	17 (28.3)	167 (40.4)	4 (8.5)	51 (11.9)
Stable disease^a^	17 (28.3)	142 (34.4)	12 (25.5)	207 (48.5)
Progressive disease	12 (20.0)	67 (16.2)	23 (48.9)	129 (30.2)
Unknown/not evaluable^b^	14 (23.3)	27 (6.5)	8 (17.0)	36 (8.4)
Objective response rate, *n* (%)	17 (28.3)	177 (42.9)	4 (8.5)	55 (12.9)
95% CI	16.9–39.7	38.1–47.6	0.5–16.5	9.7–16.1
Median time to first objective response (months)	1.9	1.9	2.7	2.1
Range	2–13	1–15	2–9	2–15

a Defined as ⩾ 7 weeks after randomization.

b No baseline tumor assessment; no post-baseline tumor assessment; stable disease that lasted < 7 weeks; ⩾ 1 lesion not evaluable.

CI, confidence interval; CP, Child-Pugh; IIR, independent imaging review; mRECIST, modified Response Evaluation Criteria In Solid Tumors.

**Figure 1.**
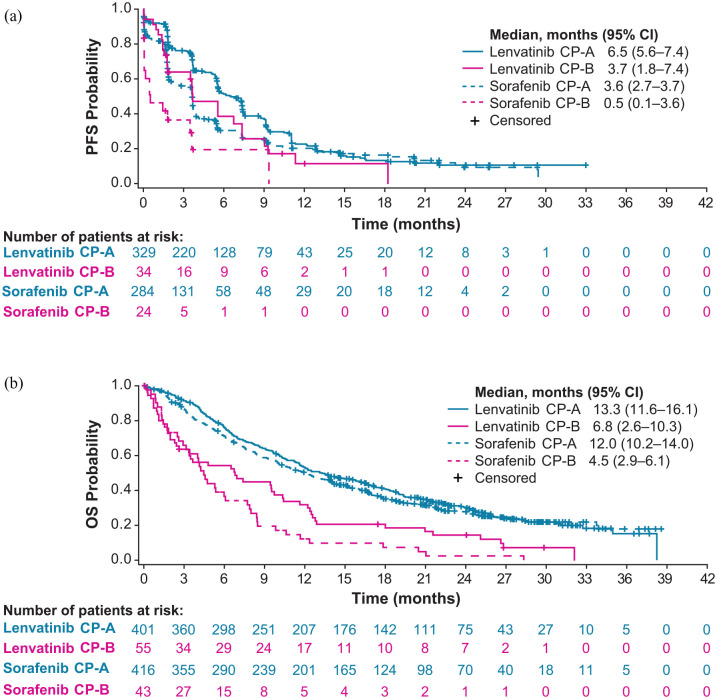
Kaplan–Meier plots of PFS by IIR per mRECIST (a) and OS (b) in lenvatinib-treated and sorafenib-treated patients based on landmark analyses after week 8 (represented as 0 on the *x*-axes). CI, confidence interval; CP, Child-Pugh; IIR, independent imaging review; mRECIST, modified Response Evaluation Criteria In Solid Tumors; OS, overall survival; PFS, progression-free survival.

Landmark analyses after week 8 showed a median PFS by IIR per mRECIST of 3.7 months (95% CI: 1.8–7.4) and median OS of 6.8 months (95% CI: 2.6–10.3) in the CP-B subgroup of patients in the lenvatinib arm (Figure 1). In the CP-A subgroup (of the lenvatinib arm), median PFS was 6.5 months (95% CI: 5.6–7.4) and median OS was 13.3 months (95% CI: 11.6–16.1). Among patients in the sorafenib arm, median PFS was 0.5 months (95% CI: 0.1–3.6) and median OS was 4.5 months (95% CI: 2.9–6.1) in the CP-B subgroup (Figure 1). In the CP-A subgroup, median PFS was 3.6 months (95% CI: 2.7–3.7) and median OS was 12.0 months (95% CI: 10.2–14.0).

### Safety

The mean lenvatinib daily dose intensity (calculated from baseline) was 8.4 mg/day (standard deviation [SD] 3.07) in the CP-B subgroup and 9.5 mg/day (SD 6.01) in the CP-A subgroup (Table 3). The mean lenvatinib daily dose intensity per starting dose in the CP-B subgroup was 6.0 mg/day (SD 2.03) in the 8 mg/day group and 9.6 mg/day (SD 2.82) in the 12 mg/day group, corresponding to 75.6 and 79.9% of the planned starting doses, respectively. The mean lenvatinib daily dose intensity per starting dose in the CP-A subgroup was 7.2 mg/day (SD 1.45) in the 8 mg/day group and 10.6 mg/day (SD 6.93) in the 12 mg/day group, corresponding to 89.6 and 88.4% of the planned doses, respectively. The median duration of lenvatinib treatment was 3.2 months (range 0.3–31.5) in the CP-B subgroup and 6.9 months (range 0–35.0) in the CP-A subgroup (Table 3). The treatment-related AE rate for grade ⩾3 episodes in the lenvatinib arm was 3.65 episodes/patient-year and 1.41 episodes/patient-year in the CP-B and CP-A subgroups, respectively (Table 3). The mean dose intensity of sorafenib in the CP-B subgroup was 653.2 mg/day (SD 165.75) and 664.8 mg/day (SD 174.23) in the CP-A subgroup, corresponding to 81.7 and 83.1% of the planned doses, respectively (Table 3). The median duration of sorafenib treatment was 1.9 months (range: 0.2–22.4) in the CP-B subgroup and 3.7 months (0.1–38.7) in the CP-A subgroup (Table 3). The treatment-related AE rate for grade ⩾3 episodes in the sorafenib arm was 3.38 episodes/patient-year and 1.71 episodes/patient-year in the CP-B and CP-A subgroups, respectively (Table 3).

**Table 3.** Safety outcomes summary^a^, adjusted by treatment duration.

Parameter	Lenvatinib		Sorafenib	
	CP-B subgroup, *n* = 60, TTD: 26.0 years	CP-A subgroup, *n* = 413, TTD: 297.9 years	CP-B subgroup, *n* = 47, TTD: 12.4 years	CP-A subgroup, *n* = 427, TTD: 226.6 years
Mean daily dose intensity, mg/day (SD)	8.4 (3.07)	9.5 (6.01)	653.2 (165.75)	664.8 (174.23)
Median duration of treatment, months (range)	3.2 (0.3–31.5)	6.9 (0–35.0)	1.9 (0.2–22.4)	3.7 (0.1–38.7)
Any treatment-related AE episodes, adjusted by patient-years^a^, *n* (AE rate^b^)	478 (18.36)	3060 (10.27)	248 (19.93)	2617 (11.55)
Grade ⩾3 episodes	95 (3.65)	419 (1.41)	42 (3.38)	388 (1.71)
Any serious TEAE episodes, adjusted by patient-years, *n* (AE rate^b^)	108 (4.15)	293 (0.98)	45 (3.62)	185 (0.82)
Treatment-related AEs leading to study drug, *n*^c^ (AE rate^b^):				
Withdrawal	15 (0.58)	35 (0.12)	5 (0.40)	37 (0.16)
Dose reduction	46 (1.77)	227 (0.76)	20 (1.61)	212 (0.94)
Interruption	59 (2.27)	275 (0.92)	18 (1.45)	235 (1.04)
Dose reduction or interruption	89 (3.42)	421 (1.41)	33 (2.65)	389 (1.72)

a AEs were graded using Common Terminology Criteria for Adverse Events version 4.0.

b Number of AE episodes per patient-year.

c Patients may be counted in >1 sub-category.

AE, adverse event; CP, Child-Pugh; SD, standard deviation; TEAE, treatment-emergent adverse event; TTD, total treatment duration.

Among patients in the lenvatinib arm, the most common treatment-emergent adverse events (TEAEs) of any grade in the CP-B subgroup were decreased appetite and hypertension (45% each), diarrhea (36.7%), and blood bilirubin increased (35.0%); the most common TEAEs of any grade in the CP-A subgroup were hypertension (41.9%), diarrhea (39.2%), and decreased appetite (32.2%; Table 4). The TEAEs most frequently leading to lenvatinib dose reduction or interruption in the CP-B subgroup were hepatic encephalopathy (15%), decreased appetite (13.3%), and blood bilirubin increased (11.7%); TEAEs most frequently leading to lenvatinib dose reduction or interruption in the CP-A subgroup were diarrhea (7.3%), decreased appetite, and proteinuria (6.8% each), and hypertension (6.5%; Supplemental Table 1).

**Table 4.** Most common TEAEs^a^ in ⩾20% of patients treated with lenvatinib.

Preferred term, *n* (%)	CP-B, *n* = 60		CP-A, *n* = 413	
	Any grade	Grade ⩾3	Any grade	Grade ⩾3
Ascites	20 (33.3)	8 (13.3)	47 (11.4)	9 (2.2)
Blood bilirubin increased	21 (35.0)	11 (18.3)	50 (12.1)	20 (4.8)
Constipation	13 (21.7)	0	61 (14.8)	3 (0.7)
Decreased appetite	27 (45.0)	7 (11.7)	133 (32.2)	15 (3.6)
Diarrhea	22 (36.7)	4 (6.7)	162 (39.2)	16 (3.9)
Dysphonia	16 (26.7)	0	96 (23.2)	1 (0.2)
Fatigue	18 (30.0)	6 (10.0)	123 (29.8)	12 (2.9)
Hepatic encephalopathy	17 (28.3)	10 (16.7)	20 (4.8)	13 (3.1)
Hypertension	27 (45.0)	14 (23.3)	173 (41.9)	97 (23.5)
Hypoalbuminemia	12 (20.0)	1 (1.7)	31 (7.5)	2 (0.5)
Hypothyroidism	15 (25.0)	0	63 (15.3)	0
Nausea	14 (23.3)	2 (3.3)	78 (18.9)	2 (0.5)
Palmar-plantar erythrodysesthesia syndrome	12 (20.0)	0	116 (28.1)	14 (3.4)
Platelet count decreased	13 (21.7)	6 (10.0)	74 (17.9)	20 (4.8)
Proteinuria	15 (25.0)	4 (6.7)	102 (24.7)	23 (5.6)
Vomiting	13 (21.7)	3 (5.0)	63 (15.3)	3 (0.7)
Weight decreased	18 (30.0)	4 (6.7)	129 (31.2)	32 (7.7)

a AEs were graded using Common Terminology Criteria for Adverse Events version 4.0.

CP, Child-Pugh; TEAEs, treatment-emergent adverse events.

In the sorafenib arm, the most common TEAEs of any grade in the CP-B subgroup were fatigue (40.4%), blood bilirubin increased, decreased appetite, and diarrhea (38.3% each), and palmar-plantar erythrodysesthesia syndrome (34.0%); the most common TEAEs of any grade in the CP-A subgroup were palmar-plantar erythrodysesthesia syndrome (54.6%), diarrhea (47.3%), and hypertension (31.9%; Table 5). The most frequent TEAEs leading to sorafenib dose reduction or interruption in the CP-B subgroup were palmar-plantar erythrodysesthesia syndrome (14.9%) and aspartate aminotransferase increased, decreased appetite, diarrhea, and fatigue (6.4% each; Supplemental Table 2). In the CP-A subgroup, the TEAEs most frequently leading to sorafenib dose reduction or interruption were palmar-plantar erythrodysesthesia syndrome (19.0%) and diarrhea (7.5%; Supplemental Table 2).

**Table 5.** Most common TEAEs^a^ in ⩾20% of patients treated with sorafenib.

Preferred term, *n* (%)	CP-B, *n* = 47		CP-A, *n* = 427	
	Any grade	Grade ⩾3	Any grade	Grade ⩾3
Abdominal pain	11 (23.4)	2 (4.3)	76 (17.8)	11 (2.6)
Alopecia	2 (4.3)	0	117 (27.4)	0
Aspartate aminotransferase increased	12 (25.5)	9 (19.1)	68 (15.9)	29 (6.8)
Blood bilirubin increased	18 (38.3)	9 (19.1)	45 (10.5)	14 (3.3)
Constipation	11 (23.4)	0	41 (9.6)	0
Decreased appetite	18 (38.3)	0	109 (25.5)	6 (1.4)
Diarrhea	18 (38.3)	2 (4.3)	202 (47.3)	18 (4.2)
Fatigue	19 (40.4)	6 (12.8)	100 (23.4)	11 (2.6)
Hypertension	8 (17.0)	4 (8.5)	136 (31.9)	64 (15.0)
Palmar-plantar erythrodysesthesia syndrome	16 (34.0)	2 (4.3)	233 (54.6)	52 (12.2)
Weight decreased	10 (21.3)	1 (2.1)	96 (22.5)	13 (3.0)

a AEs were graded using Common Terminology Criteria for Adverse Events version 4.0.

CP, Child-Pugh; TEAEs, treatment-emergent adverse events.

## Discussion

The efficacy and treatment benefit of lenvatinib in patients with uHCC and deteriorating liver function are not well established, and studies that investigate whether and how to continue treating patients with HCC whose liver function has deteriorated during treatment are lacking. In a prior post hoc analysis of REFLECT, patients with a baseline ALBI grade of 1 who received lenvatinib appeared to have a slower rate of deterioration to CP-B compared with those who received sorafenib.^18^ Among patients who had a baseline ALBI grade of 2, no major differences were observed—the median time to deterioration to CP-B was 19.9 months (95% CI: 12.1–32.0) in the lenvatinib treatment arm and 17.0 months (95% CI: 12.3–27.7) in the sorafenib treatment arm. Patients with a baseline ALBI grade of 2 had faster deterioration of liver function than those with a baseline ALBI grade of 1 in both treatment arms.

Although CP-A is generally used as an inclusion criterion in phase III trials, some patients may deteriorate to CP-B on treatment without evidence of progressive disease. Moreover, because of the lack of studies that assess the safety and effectiveness of systemic therapies in patients with early deterioration of liver function, we undertook the current study to retrospectively analyze the efficacy and safety of lenvatinib in patients whose liver function deteriorated to CP-B within 8 weeks after the start of treatment. It is important to note that these patients could have deteriorated to CP-B for several reasons, including progression of the underlying liver cirrhosis, toxicity of the anti-tumor treatment, as well as tumor progression. Determining the exact cause is challenging given that a combination of factors for every patient can contribute to liver function deterioration. The results from our study showed that patients in the lenvatinib arm had a similar median tumor size reduction of approximately 16% from baseline to week 8, irrespective of whether patients had early progression to CP-B or remained CP-A within the first 8 weeks of treatment; thus, tumor progression was not a likely cause of progression to CP-B liver function. The similarity in tumor size reduction among the CP-B and CP-A subgroups from baseline to week 8 also supports the efficacy of lenvatinib treatment in the overall population of patients with uHCC. Precise reasons for progression to CP-B status could not be determined; notably, however, most patients in the CP-A subgroup who received lenvatinib entered the study with ALBI grade 1, while most patients in the CP-B subgroup who received lenvatinib entered the study with ALBI grade 2 (73.3% of patients who progressed to CP-B status were classified as ALBI grade 2 at baseline). Notably, a larger percentage of patients who received sorafenib and progressed to CP-B status on treatment had an ECOG PS of 1 and were BCLC stage C at baseline (61.7 and 95.7%, respectively) than patients who received lenvatinib and progressed to CP-B status (33.3 and 76.7%, respectively), which may have influenced results.

Among patients who progressed to CP-B, lenvatinib showed promising efficacy. The ORR for these patients was 28.3% (no CRs were observed). Moreover, responses occurred relatively quickly, as the median time to first objective response was 1.9 months in this subgroup. Based on the landmark analyses, the median OS was 6.8 months starting at week 8 and median PFS was 3.7 months starting at week 8 in patients from the CP-B subgroup of the lenvatinib arm (conversely, median OS was 4.5 months starting at week 8 and median PFS was only 0.5 months starting at week 8 in patients from the CP-B subgroup of the sorafenib arm, possibly suggesting more limited antitumor activity). The median duration of lenvatinib treatment was 3.2 months in the CP-B subgroup. Importantly, no new safety signals were observed. Taken together, these data suggest that lenvatinib may continue to be beneficial in patients with early progression to CP-B liver function during treatment and support its efficacy in the overall population of patients with uHCC. These results are consistent with the CELESTIAL trial of patients with advanced HCC, which demonstrated median OS and PFS of 8.5 and 3.7 months, respectively, in patients who received cabozantinib and progressed to CP-B liver function by week 8 of treatment (both outcomes were analyzed from randomization).^4^ These results suggest that continuation of systemic therapies may be a viable option for these patients, who have poor prognosis and limited treatment options.

Caution should be used when directly comparing subgroups (i.e. CP-B and CP-A subgroups between lenvatinib and sorafenib treatment arms) in this study, because the groups are based on a post-baseline status. Thus, all comparisons are descriptive in nature, as no statistical comparisons can be made. As expected, outcomes (i.e. PFS from week 8, OS from week 8, and ORR) for patients who remained CP-A within the first 8 weeks of treatment were better compared to those who converted to CP-B liver function. Regardless of liver function classification, these outcomes support the use of lenvatinib in uHCC. Notably, the mean lenvatinib daily dose intensity in patients who progressed to CP-B liver function (8.4 mg) was consistent with the maximum tolerable lenvatinib dose in patients with advanced HCC and CP-B liver function shown in a phase I dose-escalation study (8 mg).^19^ In addition, the mean lenvatinib daily dose was slightly lower in the CP-B group (6.0 mg in the 8 mg/day group and 9.6 mg in the 12 mg/day group) than in the CP-A group (7.2 mg in the 8 mg/day group and 10.6 mg in the 12 mg/day group), indicating that clinical benefits can be observed despite increased dose reductions in patients with early liver function deterioration. Additional studies must be completed to determine the effectiveness of systemic therapies in CP-B patients. Furthermore, studies comparing outcomes in patients with on-treatment liver function deterioration who continued to receive lenvatinib *versus* those who discontinued lenvatinib would provide further insights. However, these efficacy results, combined with a manageable safety profile, suggest that treatment with lenvatinib may be considered in patients who progress to CP-B liver function.

## Supplemental Material

sj-docx-1-tam-10.1177_17588359221116608 – Supplemental material for Lenvatinib in patients with unresectable hepatocellular carcinoma who progressed to Child-Pugh B liver function Click here for additional data file. Supplemental material, sj-docx-1-tam-10.1177_17588359221116608 for Lenvatinib in patients with unresectable hepatocellular carcinoma who progressed to Child-Pugh B liver function by Jasmine Huynh, May Thet Cho, Edward Jae-Hoon Kim, Min Ren, Zahra Ramji and Arndt Vogel in Therapeutic Advances in Medical Oncology

## Declarations

**Ethics approval and consent to participate:** All relevant institutional review boards approved the REFLECT study, which was done in accordance with the Declaration of Helsinki and local laws. All patients provided written informed consent.

**Consent for publication:** Not applicable.

**Availability of data and materials:** The data will not be available for sharing at this time because the data are commercially confidential. However, Eisai will consider written requests to share the data on a case-by-case basis.
