# Supplementary material for: Lenvatinib in patients with unresectable hepatocellular carcinoma who progressed to Child-Pugh B liver function
Source: Ther Adv Med Oncol. 2022 Aug 24;14:17588359221116608. doi: 10.1177/17588359221116608 (PMC9425881; doi:10.1177/17588359221116608)
Supplement: sj-docx-1-tam-10.1177_17588359221116608 – Supplemental material for Lenvatinib in patients with unresectable hepatocellular carcinoma who progressed to Child-Pugh B liver function [file sj-docx-1-tam-10.1177_17588359221116608.docx]

**Supplementary Table 1.** Most Common TEAEs Leading to Dose Reduction or Interruption (Incidence ≥4% in any Group) in Patients Treated With Lenvatinib

| **Preferred term, n (%)** | **Child**–**Pugh B**  **n = 60** | **Child**–**Pugh A**  **n = 413** |
| --- | --- | --- |
| Ascites | 4 (6.7) | 6 (1.5) |
| Blood bilirubin increased | 7 (11.7) | 13 (3.1) |
| Decreased appetite | 8 (13.3) | 28 (6.8) |
| Diarrhea | 6 (10.0) | 30 (7.3) |
| Dyspnea | 3 (5.0) | 5 (1.2) |
| Fatigue | 3 (5.0) | 24 (5.8) |
| Hepatic encephalopathy | 9 (15.0) | 11 (2.7) |
| Hypertension | 2 (3.3) | 27 (6.5) |
| Palmar-plantar erythrodysesthesia syndrome | 1 (1.7) | 24 (5.8) |
| Platelet count decreased | 4 (6.7) | 18 (4.4) |
| Proteinuria | 5 (8.3) | 28 (6.8) |
| Weight decreased | 3 (5.0) | 17 (4.1) |

TEAEs, treatment-emergent adverse events.

**Supplementary Table 2.** Most Common TEAEs Leading to Dose Reduction or Interruption (Incidence ≥4% in any Group) in Patients Treated With Sorafenib

| **Preferred term, n (%)** | **Child**–**Pugh B**  **n = 47** | **Child**–**Pugh A**  **n = 427** |
| --- | --- | --- |
| Abdominal pain | 2 (4.3) | 6 (1.4) |
| Anemia | 2 (4.3) | 6 (1.4) |
| Aspartate aminotransferase increased | 3 (6.4) | 15 (3.5) |
| Decreased appetite | 3 (6.4) | 12 (2.8) |
| Diarrhea | 3 (6.4) | 32 (7.5) |
| Fatigue | 3 (6.4) | 14 (3.3) |
| Hepatic encephalopathy | 2 (4.3) | 1 (0.2) |
| Hepatic function abnormal | 2 (4.3) | 7 (1.6) |
| Hypertension | 1 (2.1) | 17 (4.0) |
| Jaundice cholestatic | 2 (4.3) | 0 (0.0) |
| Palmar-plantar erythrodysesthesia syndrome | 7 (14.9) | 81 (19.0) |

TEAEs, treatment-emergent adverse events.
